# Comprehensive and Site-Specific Characterization of Protein N‑Glycosylation in AD Samples Reveals Its Potential Roles in Protein Aggregation and Synaptic Dysfunction

**DOI:** 10.1021/acs.analchem.5c02455

**Published:** 2025-10-03

**Authors:** Xing Xu, Haiyan Tan, Kejun Yin, Senhan Xu, Zeyu Wang, Geidy E. Serrano, Thomas G Beach, Xusheng Wang, Junmin Peng, Ronghu Wu

**Affiliations:** † School of Chemistry and Biochemistry and the Petit Institute for Bioengineering and Bioscience, 1372 Georgia Institute of Technology, Atlanta, Georgia 30332, United States; ‡ Center for Proteomics and Metabolomics, St. Jude Children’s Research Hospital, Memphis, Tennessee 38105, United States; § Banner Sun Health Research Institute, Sun City, Arizona 85351, United States; ∥ Department of Neurology, University of Tennessee Health Science Center, Memphis, Tennessee 38163, United States; ⊥ Department of Structural Biology and Department of Developmental Neurobiology, St. Jude Children’s Research Hospital, Memphis, Tennessee 38105, United States

## Abstract

Alzheimer’s disease (AD) is a neurodegenerative disorder characterized by progressive cognitive decline. Emerging evidence strongly suggests that protein glycosylation is strongly related to this disease. However, the extent and functional consequences of site-specific N-glycosylation alterations in AD remain to be further explored. Here, we employed a dendrimer boronic acid (DBA)-based enrichment strategy combined with multiplexed proteomics to systematically analyze protein N-glycosylation in post-mortem human brain tissues. We identified 3,105 N-glycosylation sites on 1,299 glycoproteins from nine AD cases and nine healthy controls, and performed a systematic and site-specific investigation of glycosylation alterations in AD. Glycoproteins involved in cholesterol efflux were upregulated, whereas those associated with chemical synaptic transmission and ion transport were significantly downregulated in AD compared to control brain samples. We observed widespread dysregulation of N-glycosylation across multiple protein domains, particularly in the ConA-like lectins/glucanases and Zn-dependent exopeptidases domains. Notably, we identified 161 N-glycosylation sites located within aggregation-prone regions (APRs), and reduced glycosylation at APRs on plaque-associated glycoproteins may be associated with protein aggregation and plaque formation. Additionally, downregulated N-glycosylation sites were enriched in synaptic membrane proteins, such as Ca^2+^ ion channels, GABA-gated chloride channels, and glutamate receptors, implicating glycosylation loss in synaptic dysfunction. Our findings suggest that the loss of N-glycosylation may contribute to the pathogenesis of AD through impairing synaptic transmission and promoting protein aggregation. This study provides novel insights into glycosylation-dependent mechanisms of neurodegeneration, highlighting N-glycosylation as a potential therapeutic target for AD treatment.

## Graphical abstract

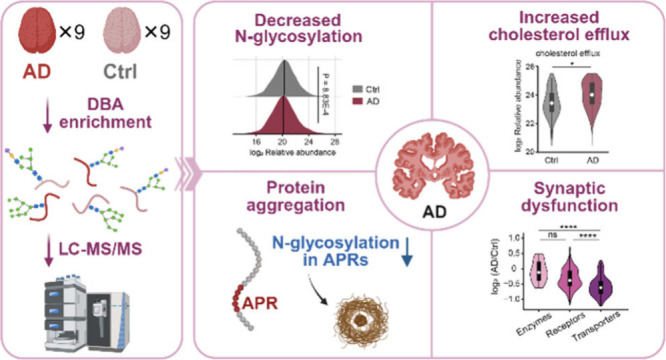

## Introduction

AD is a progressive neurodegenerative disorder characterized by synaptic dysfunction, protein aggregation, and widespread neuronal loss. As a leading cause of dementia and mortality worldwide, AD currently lacks effective preventive or curative treatments.^1^ The defining hallmarks of AD pathology are the accumulation of amyloid-beta (Aβ) plaques and neurofibrillary tangles composed of hyperphosphorylated tau, which disrupt cellular homeostasis and impair cognitive function. Despite extensive research, the molecular changes underlying these pathological features remain incompletely understood. Increasing evidence suggests that protein modifications, particularly N-glycosylation, play a crucial role in modulating protein stability and aggregation, and synaptic function in AD.^2–7^ However, site-specific analysis of the extent and functional consequences of N-glycosylation alterations in AD remains to be further explored.

N-glycosylation is a highly conserved protein modification that involves the attachment of glycan to the asparagine residue within the consensus sequence N-X-S/T (where X is any amino acid except proline). This modification is essential for protein folding, trafficking, stability, and interactions.^8–11^ In the central nervous system, N-glycosylation regulates key processes such as synaptic transmission, ion channel activity, receptor function, and immune signaling. Notably, many proteins implicated in AD, including amyloid precursor protein (APP), and neurotransmitter receptors, are also glycosylated, suggesting that dysregulation of glycosylation may contribute to disease pathology. A recent study has highlighted the potential neuroprotective role of N-glycosylation in preventing protein aggregation.^12^ Glycans can shield aggregation-prone regions (APRs) by sterically hindering intermolecular interactions, thereby reducing the propensity for protein misfolding and aggregation. However, whether alterations in N-glycosylation directly contribute to the aggregation of plaque-associated proteins in AD remains unclear. Furthermore, while previous proteomic studies have identified broad changes in protein abundance in AD brains, the specific alterations in N-glycosylation at individual sites and their functional implications at the synapse remain poorly understood. Given that synaptic dysfunction is an early event in AD, elucidating N-glycosylation changes in synaptic glycoproteins may provide critical insights into disease mechanisms.

In this work, we employed a Dendrimer-conjugated Boronic Acid derivative (DBA) enrichment strategy combined with multiplexed proteomics to comprehensively quantify N-glycosylation changes in post-mortem human brain tissues, providing clinically relevant insights beyond transgenic mouse models.^13^ We identified 3,105 N-glycosylation sites on 1,299 glycoproteins from nine AD tissue samples and nine healthy controls (sample information in Table S1). Glycoproteins involved in cholesterol efflux were upregulated, while those associated with chemical synaptic transmission and ion transport were downregulated. Quantitative glycoproteomics combined with functional annotation revealed widespread dysregulation of N-glycosylation across multiple protein domains, APRs and synaptic regions. These alterations suggest that glycosylation loss may contribute to protein aggregation, synaptic dysfunction, and impaired neurotransmission in AD. Our study provides a detailed map of N-glycosylation changes, offering new insights into AD pathogenesis and identifying potential therapeutic targets.

## Methods

### Brain Sample Preparation and Protein Extraction

Human post-mortem brain frontal cortex samples were provided by the Brain and Body Donation Program (www.brainandbodydonationprogram.org) at Banner Sun Health Research Institute.^14^ Clinical and pathological diagnoses were based on the established criteria.^14^ The frozen AD or control brain samples were lysed as previously described with slight modification.^15^ Detailed procedures can be found in the Supporting Information.

### Enrichment of Glycopeptides Using DBA

Glycopeptides were enriched using the DBA enrichment method developed in our lab previously.^16^ Peptides from the AD and control samples were dissolved in the binding buffer (DMSO, 0.5% triethylamine) and incubated for 30 min with the DBA magnetic beads at room temperature. After the incubation, the beads were washed with the washing buffer (50% H_2_O, 50% DMSO, 100 mM NH_4_OAc, pH = 10) four times. Then, enriched glycopeptides were eluted twice through the incubation with a solution containing acetonitrile: water: trifluoroacetic acid (50:49:1) at room temperature for 1 h. The enriched samples were dried in a lyophilizer overnight for the subsequent TMT labeling and the PNGase F treatment.

### TMT Labeling and PNGase F Treatment

Dried glycopeptides in each sample were labeled with each channel of the multiplexed TMT reagents (Thermo) following the published protocol.^17^ The labeled peptide or glycopeptide solutions were mixed and then purified using the tC18 Sep-Pak cartridges and dried overnight. The PNGase F treatment was carried out by dissolving TMT-labeled peptides in 40 μL of 40 mM ammonium bicarbonate (pH = 9) (Sigma-Aldrich) in heavy-oxygen water (H_2_^18^O), followed by the addition of 3 μL of 1 unit/μL PNGase F (Sigma-Aldrich). The sample was shaken at 37 °C for 3 h. The reaction was quenched with formic acid (FA) until the pH reached ∼2.0. The samples were desalted again with the stage-tip method and dried overnight.

### High-pH HPLC Fractionation and LC-MS/MS Analysis

The mixed TMT labeled sample was fractionated into 25 fractions using high-pH HPLC. Then, the sample was dissolved in 6 μL solvent with 5% ACN and 4% FA, and 4 μL of the solution were loaded by a Dionex WPS-3000TPLRS autosampler onto a microcapillary column packed with C18 beads. Peptides were separated using a nanoflow reversed phase HPLC. The microcapillary column was directly coupled for MS analysis using a Nanospray Flex ion source. The peptides were analyzed using an Orbitrap Exploris 480 mass spectrometer (Thermo) (detailed information in the Supporting Information).

## Results

### Global Analysis of N-Glycoproteins in Human Brains Using Synergistic Boronic Acid–Glycan Interactions

Previous studies demonstrated alterations in sugar metabolism in AD brains compared to healthy controls.^18–20^ However, how these metabolic changes influence protein glycosylation in AD remains to be explored, especially at the site-specific level. Given the typically low abundance of glycoproteins, we employed an enrichment strategy developed in our laboratory,^16^ utilizing synergistic and reversible covalent interactions between DBA and glycans for ultrasensitive glycoproteomic analysis. For quantitative profiling, glycopeptides enriched from both AD and control samples were labeled with TMT18 for comparative analysis. All samples were subsequently treated with PNGase F in heavy-oxygen water (H_2_^18^O), introducing a unique isotopic tag at the glycosylation site for MS analysis (Figure 1A). This approach enabled the identification of 3,105 N-glycosylation sites from 1,299 glycoproteins across 18 samples (Figure S1; Table S2 and S3). To account for potential variations in protein abundance across samples, whole-protein abundance normalization was applied.

**Figure 1:**
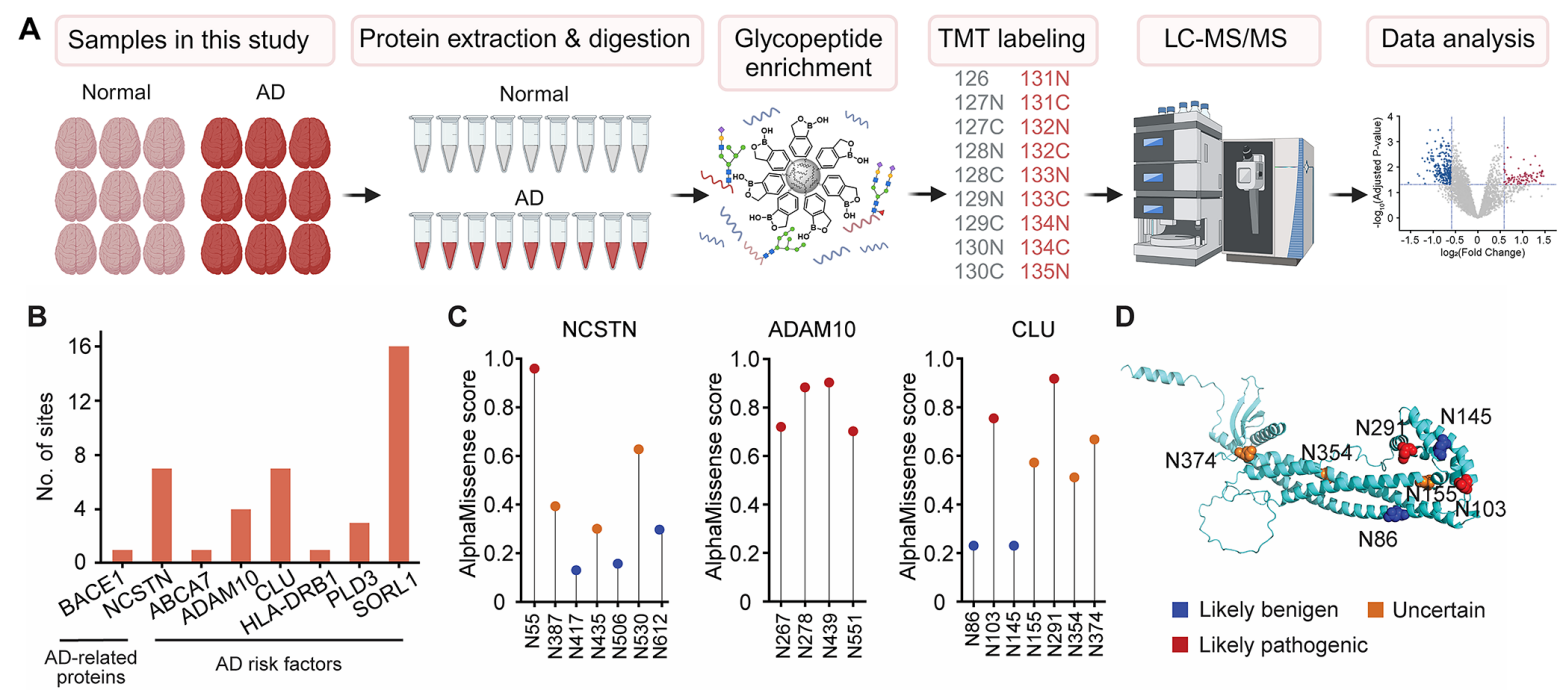
Comprehensive identification of N-glycoproteins from human brains. (A) Schematic experimental workflow. Proteins from nine normal and nine symptomatic AD human brains were first extracted. Glycopeptides were enriched using the DBA beads and then labeled with the TMT18 reagents, respectively. Enriched glycopeptides were treated with PNGase F in H_2_^18^O before LC-MS/MS analysis. (B) Bar graph showing the number of N-glycosylation sites identified on AD-related proteins and GWAS-identified AD risk factors. (C) AlphaMissense pathogenicity scores for N-glycosylation sites in NCSTN, ADAM10, and CLU. (D) Glycosylated asparagine residues highlighted as spheres in the AlphaFold-predicted structure of CLU. The color of each site represents its predicted pathogenicity level.

Functional mapping of tissue expression using FUMA analysis^21^ revealed that glycoproteins are enriched in several brain regions, including the cortex, hypothalamus, hippocampus, and amygdala (Figure S2). Gene ontology (GO) analysis based on biological processes and molecular functions indicated that the enriched glycoproteins are involved in a variety of critical functions, including immune system processes (*P* = 1.85 × 10^–27^), exocytosis (*P* = 4.1 × 10^–21^), regulation of neurogenesis (*P* = 6.37 × 10^–21^), synaptic signaling (*P* = 1.24 × 10^–26^), calcium ion binding (*P* = 1.78 × 10^–21^), cell adhesion molecule binding (*P* = 1.36 × 10^–26^), and gated channel activity (*P* = 1.51 × 10^–22^) (Figure S3).

### N-Glycosylation in AD-Related Proteins and AD Risk Factors

Comparison of the N-glycoproteome between the mouse and human brain tissues revealed substantial differences, indicating that the mouse brain may not be an ideal model for studying human brain N-glycosylation (Figure S4).^22^ Analysis of the human brain N-glycoproteome revealed that approximately 40% of glycoproteins carried one N-glycosylation site, over 24% contained two sites, and nearly 35% harbored more than two sites (Figure S5A). Regarding the consensus sequence motifs, 56% of N-glycosylation sites followed the N-X-T motif, 43% conformed to N-X-S, and only approximately 1% matched the N-X-C motif, consistent with a previous finding (Figure S5B).^5^

Importantly, our data set captured AD-related proteins, including BACE1 and NCSTN, as well as AD genetic risk factors identified through genome-wide association studies (GWAS), such as ABCA7, ADAM10, CLU, HLA-DRB1, PLD3, and SORL1 (Figure 1B). Pathogenicity prediction using AlphaMissense^23^ showed high-risk N-glycosylation sites, such as N55 in NCSTN, four sites in ADAM10 (N267, N278, N439, N551), and N103 and N291 in PLD3 (Figure 1C–D). These results suggest that the mutations affecting N-glycosylation sites in AD-related proteins and genetic risk factors may contribute to AD pathogenesis, highlighting the potential of N-glycosylation as a mechanistic link between genetic risk and disease progression.

### Dysregulation of N-Glycoproteome in AD Brains

Quantification of glycoproteins was performed via multiplex proteomics using the TMT labeling, and 1,041 N-glycoproteins were quantified (Table S4). One control sample, which showed characteristics similar to the AD group, was statistically identified as an outlier and excluded from further analysis (Figure 2A). To investigate how the N-glycoproteome changes in AD, we first compared the relative abundance of glycopeptides between the AD and control groups (Figure 2B and S6). The overall abundance of the N-glycoproteome in AD was slightly lower than that in controls, which may be attributed to changes in sugar metabolism in the AD brains.^18^ Differential analysis identified 501 significantly altered glycopeptides (|log2FC| > 0.5 and *P* < 0.05), with 58 upregulated and 127 downregulated glycoproteins (Figure 2C and Table S5). Notably, several previously identified AD markers were also found to be differentially expressed in this work, including PLP1,^24^ NEFM,^25^ AQP1,^26^ and TNC.^27^ Additionally, we identified other differentially expressed glycoproteins, such as TSN8, CD38, and FRZB.

**Figure 2:**
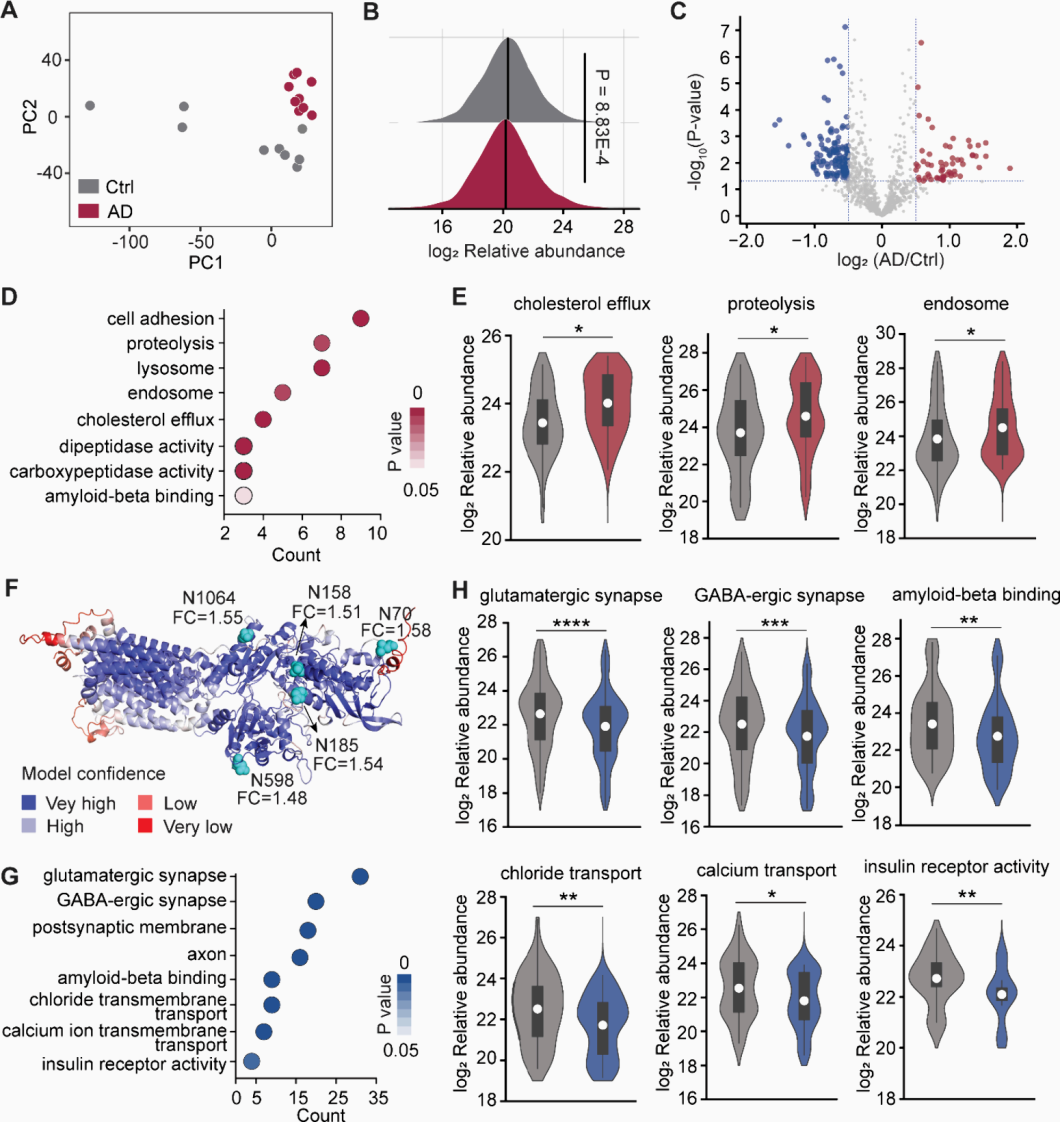
Quantitative analysis of the N-glycoproteome in AD. (A) Principal-component analysis (PCA) of N-glycoproteins from the AD and control samples. (B) Distributions of log_2_ relative abundance of glycopeptides in the AD versus control samples. (C) Volcano plot showing differentially regulated glycoproteins in AD. (D) Representative function annotations of upregulated glycoproteins in AD, obtained from the DAVID database. Colors indicate enrichment significance. (E) Violin plots showing log_2_ relative abundance of glycoproteins between AD and control in the indicated GO terms for the upregulated glycoprotein group. (F) Glycosylated asparagine residues highlighted as cyan spheres in the AlphaFold-predicted structure of NPC1. (G) Representative function annotations of downregulated glycoproteins in AD, obtained from the DAVID database. (H) Violin plots showing log_2_ relative abundance of glycoproteins between AD and control in the indicated GO terms among the downregulated glycoproteins. Interquartile ranges (IQRs) are shown as boxes, with the median as a white dot and the whiskers extending up to the most extreme points within 1.5-fold IQR. P values are assessed using the two-sided *t* test: * *P* < 0.05, ** *P* < 0.01, *** *P* < 0.001, **** *P* < 0.0001.

GO analysis of the 58 significantly upregulated glycoproteins revealed strong associations with proteolysis (CPQ, CPM, CNDP1, ADAM10/11, etc.), lysosome (FUCA2, CRYAB, NAGLU, etc.), endosome (LRP2, CD9, PICALM, etc.), and cholesterol efflux (ABCA2/8, LIPA, NPC1, etc.) (Figure 2D and 2E). These glycoproteomic results are consistent with previous studies highlighting impaired cholesterol homeostasis in AD.^28–30^ Cholesterol plays a crucial role in regulating both the production and clearance of amyloid-beta (Aβ), with elevated cholesterol levels shown to increase Aβ accumulation in cellular and most animal models of AD.^29^ Among the key regulators of intracellular cholesterol homeostasis, NPC intracellular cholesterol transporters (NPC1 and NPC2) and low-density lipoprotein receptors (LRP1, LRP1B, LRP2, LRP4, and LRP11) are essential for maintaining cholesterol balance. Notably, N-glycosylation of NPC1 and LRP2 were significantly elevated in the AD brain tissues, whereas other cholesterol regulators remained unchanged or were downregulated (Figure 2F; Figure S7). These findings suggest that the dysregulation of N-glycosylation in NPC1 and LRP2 may contribute to cholesterol homeostasis disruptions in AD.

Conversely, among 127 significantly downregulated glycoproteins, those associated with glutamatergic synapses (GRIN1/2A, GRM2/5, etc.), GABA-ergic synapses (GABRA1/2/4, GABBR1, GABRD etc.), and axon function are enriched. The results are aligned well with previous reports of widespread synapse loss and neuronal damage in AD brains.^31–34^ Furthermore, glycoproteins involved in chloride and calcium transport were also downregulated. Given that synaptic ion channels play a fundamental role in facilitating and fine-tuning synaptic transmission, their loss disrupts membrane potential, ultimately impairing neural network activity (Figure 2G−H). We also identified three glycoproteins associated with Aβ binding that were upregulated (ITGA2, PICALM, and CRYAB), while nine glycoproteins involved in Aβ binding were downregulated (CACNA1A, EPHA4, GRIA2, GRIA3, GRIA4, GRIN1, GRIN2A, GRIN2B, and HSPG2). These findings suggest a reduced capacity for Aβ clearance in the AD brains. Collectively, our study provides novel insights into synaptic alterations in AD at the protein modification level, enhancing our understanding of the molecular mechanisms of the disease.

**Figure 3:**
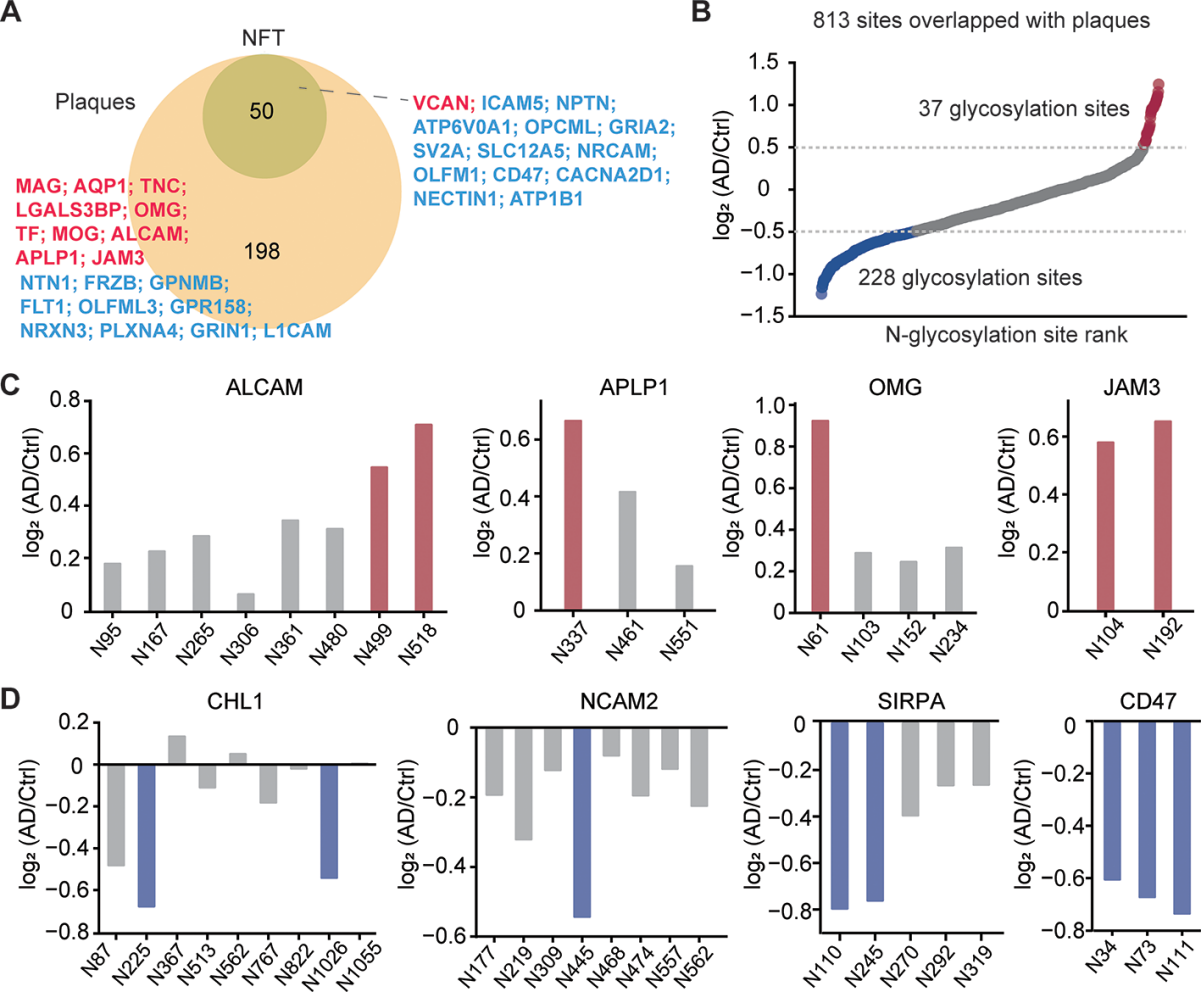
Analysis of plaque- and NFT-associated glycoproteins. (A) Number of glycoproteins whose parent proteins colocalized with Aβ plaques and NFTs. Text colors indicate whether glycoproteins are upregulated (red) or downregulated (blue) in Aβ plaques or NFTs. (B) Distribution of log_2_FC in N-glycosylation from plaque-associated glycoproteins. (C) and (D) Examples of glycosylation sites that were upregulated (C) or downregulated (D) in AD.

### Identification of Neuropathology-Associated Glycoproteins

Aβ plaques and NFTs contain numerous proteins in addition to Aβ and tau.^35^ Investigating plaque- and NFT-associated proteins is critical for understanding the factors contributing to their formation. The NeuroPro database includes 2,199 proteins, with 557 proteins identified as being present in or enriched within Aβ plaques or NFTs.^35^ The overlap between plaque- and NFT-associated proteins suggests that many proteins abundant in tau-rich dystrophic neurites are also present in neuritic plaques (Figure S8). To further elucidate the relationship between protein N-glycosylation and the hallmarks of the AD neuropathologies (Aβ plaques and NFTs), we examined the overlap between glycoproteins and proteins previously identified as colocalized with Aβ plaques and NFTs. Our analysis identified 248 plaque-associated glycoproteins and 50 NFT-associated glycoproteins (Table S6). Notably, all NFT-associated glycoproteins were also abundant in plaques (Figure 3A). This finding may be explained by the extracellular nature of Aβ plaques, as glycoproteins are predominantly localized in extracellular regions. In contrast, NFTs, which initially form intracellularly, may persist as remnants in the extracellular space following neuronal death, leading to a lower number of NFT-associated glycoproteins. GO enrichment analysis revealed that these plaque-associated glycoproteins were significantly enriched in biological processes related to cell adhesion and axon guidance (Figure S9).

To investigate the involvement of protein N-glycosylation in the plaque formation, we examined the changes in N-glycosylation of these plaque-associated proteins (Figure 3B). Given the critical role of cell adhesion molecules in modulating interactions with amyloid beta precursor protein (APP) in AD development,^36^ we focused on this subset of glycoproteins. We identified 31 upregulated N-glycosylation sites from 9 proteins and 65 downregulated N-glycosylation sites from 20 proteins involved in cell adhesion (Figure 3C and 3D). These glycoproteins may serve as biomarkers for AD diagnosis. For example, activated leukocyte cell adhesion molecule (ALCAM) was previously associated with age and cognitive decline severity.^37^ The N-glycosylation sites of ALCAM at N499 and N518 were upregulated in AD, whereas other N-glycosylation sites did not show significant changes. Similarly, amyloid precursor-like protein 1 (APLP1), a homologue of APP and a substrate of γ-secretase, was reported to undergo cleavage influenced by N-glycosylation.^38^ We quantified three different N-glycosylation sites of APLP1 (N337, N461, and N551) and found that the N-glycosylation site at N337 was upregulated more than 1.5-fold in AD, suggesting that glycosylation at this site may modulate γ-secretase function. Additionally, oligodendrocyte-myelin glycoprotein (OMG) exhibited upregulation of its N-glycosylation site at N61, whereas other N-glycosylation sites (N103, N152, and N234) remained unchanged. Some proteins demonstrated upregulation across all their N-glycosylation sites, including junctional adhesion molecules (JAMs) and tenascin-C (TNC) (Figure S10). These results suggest that protein N-glycosylation on these glycoproteins may enhance their interactions with the plaques.

Conversely, several adhesion molecules exhibited downregulation of specific N-glycosylation sites in AD. For example, neural cell adhesion molecule L1-like protein (CHL1) exhibited downregulation at N225 and N1026. Similarly, neural cell adhesion molecule 2 (NCAM2) has nine N-glycosylation sites, among which only the site at N445 was downregulated in AD. Given that N-glycosylation influences protein solubility,^22^ a comparative analysis of NCAM2 N-glycosylation site–specific solubility relative to the solubility of its parent protein revealed that N-glycosylation at N445 substantially enhances protein solubility compared to glycosylation at other sites (Figure S11). These findings suggest downregulation of glycosylation at these sites may decrease solubility, thereby promoting coaggregation with plaques. Notably, we also identified two N-glycosylation sites of signal regulatory protein alpha (SIRPA) at N110 and N245, as well as three N-glycosylation sites of CD47 at N34, N73, and N111, which were downregulated in AD (Figure 3D). Given that SIRPA binding to CD47 is independent of N-glycosylation (Figure S12),^39^ the observed downregulation of these sites may instead affect protein structure or solubility. These findings highlight the role of protein N-glycosylation in modulating protein solubility, therefore influencing their abundance in Aβ plaques and NFTs. By revealing alterations in N-glycosylation in AD pathogenesis, our results provide valuable insights into potential biomarkers for monitoring disease progression.

### Differential Regulation of N-Glycosylation across Protein Domains in AD Brains

To investigate site-specific alterations of N-glycosylation in AD, we analyzed singly N-glycosylated peptides from a total of 3,685 quantified glycopeptides (Table S5), and 2,724 N-glycosylation sites were quantified in the AD brain tissues (Table S7). Among these, 113 N-glycosylation sites were upregulated, while 630 sites were downregulated (Figure 4A). For example, all four N-glycosylation sites (N100, N118, N341, and N403) in ENPP6, a choline-specific phosphodiesterase involved in choline metabolism, were significantly upregulated in AD. Increased N-glycosylation of ENPP6 may contribute to an imbalance in choline homeostasis in the AD brains. Conversely, all ten N-glycosylation sites (N54, N137, N195, N214, N303, N583, N646, N764, N795, and N796) in ICAM5 were downregulated by more than 1.5-fold in AD, potentially impairing neuronal development.

**Figure 4:**
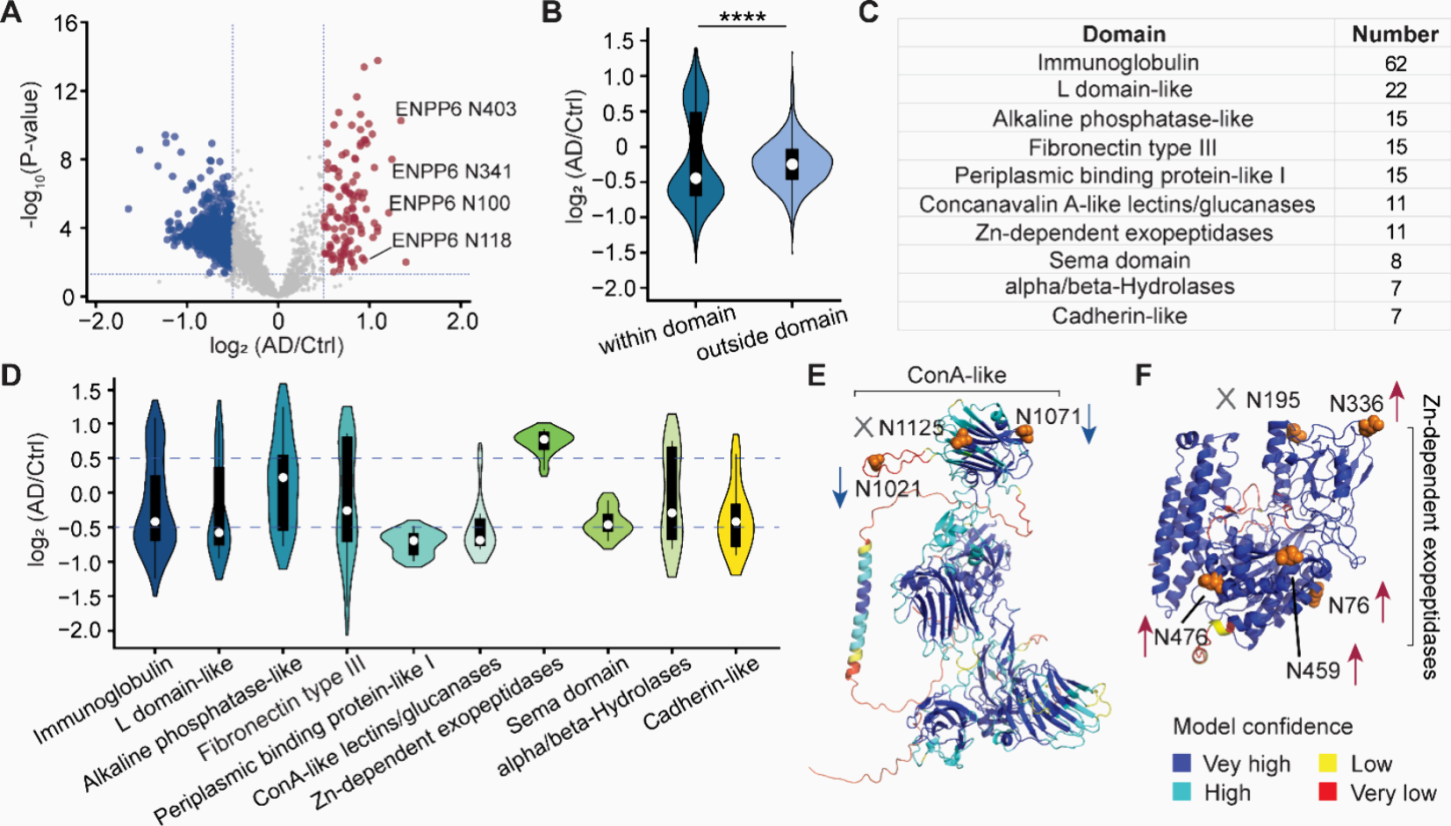
Site-specific analysis of alterations in N-glycosylation based on protein domains. (A) Volcano plot showing quantified N-glycosylation sites. (B) Comparison of abundance changes of N-glycosylation sites located within or outside of protein domains. Interquartile ranges (IQRs) are shown as boxes, with the median as a white dot and the whiskers extending up to the most extreme points within 1.5-fold IQR. P values are assessed using the two-sided *t* test: * *P* < 0.05, ** *P* < 0.01, *** *P* < 0.001, **** *P* < 0.0001. (C) Summary of protein domain types and the number of N-glycosylation sites located within each protein domain. (D) Comparison of abundance changes in N-glycosylation sites located within each protein domain type. (E) and (F) Glycosylated asparagine residues highlighted as orange spheres in the AlphaFold-predicted structure of CNTNAP5 (E) and FOLH1 (F). Arrows indicate downregulated (blue) or upregulated (red) N-glycosylation sites.

We next assessed glycosylation changes relative to protein structural features. No significant differences were observed in N-glycosylation sites located in different types of secondary structures or solvent accessibility regions (Figure S13), suggesting that AD-related glycosylation changes do not preferentially target proteins based on these structural properties. However, N-glycosylation decreases were more pronounced at sites located within protein domains compared to those outside domains (Figure 4B). Given that protein domains serve as distinct functional and structural units, these glycosylation changes may have significant functional consequences.

To elucidate domain-specific changes, we identified the ten most abundant protein domains containing N-glycosylation sites and summarized the number of glycosylation sites identified within each of these domains (Figure 4C). Additionally, we compared the log_2_FC values of N-glycosylation sites across different types of domains (Figure 4D). Glycosylation sites within the L domain-like, periplasmic binding protein-like I, and concanavalin A-like lectins/glucanases domains exhibited decreased glycosylation levels. In contrast, glycosylation sites within the Zn-dependent exopeptidase domain showed an increase in glycosylation (Figure 4D). For the concanavalin A-like/glucanase domain involved in oligosaccharide binding, we quantified changes at 11 N-glycosylation sites located within this domain, which overall decreased in AD. This finding suggests that decreased N-glycosylation in the concanavalin A-like domain may impact cell adhesion and intercellular communication in both the peripheral and central nervous systems. For example, seven N-glycosylation sites were quantified in CNTNAP5, three of which were located within the concanavalin A-like lectins/glucanases domain. Notably, N-glycosylation at N1021 and N1071 was downregulated by more than 1.5-fold (Figure 4E). Conversely, N-glycosylation sites located within the Zn-dependent exopeptidase domain were predominantly upregulated in the AD brains, suggesting increased hydrolysis activity. For instance, FOLH1, an enzyme responsible for cleaving N-acetyl-l-aspartyl-l-glutamate (NAAG) to yield free glutamate in the synaptic cleft, exhibited increased glycosylation. Among six quantified N-glycosylation sites in FOLH1, five were located within the Zn-dependent exopeptidase domain. Previous studies demonstrated that N-glycosylation was critical for NAAG-hydrolyzing activity.^40^ We found that N-glycosylation at N76, N336, N459, and N476 were upregulated by more than 1.5-fold in the AD brains (Figure 4F). N-Glycosylation dysregulation in different protein domains may potentially alter their structural integrity and functional roles.

### Identification of N-Glycosylation Sites Predicted to Prevent Protein Aggregation

Given the role of N-glycosylation in preventing protein aggregation,^12^ and protein aggregation is a common feature of neurodegenerative diseases.^41^ we analyzed all singly N-glycosylated peptides using TANGO^42^ to identify sites located within aggregation-prone regions (APRs). APRs are short, typically hydrophobic amino acid stretches that promote protein aggregation. This analysis identified 161 N-glycosylation sites localized within APRs (Table S8). Among these, 6.8% of them were located in α-helices, while 46.6% were found in coil regions and 46.6% in β-strands (Figure 5A).

**Figure 5:**
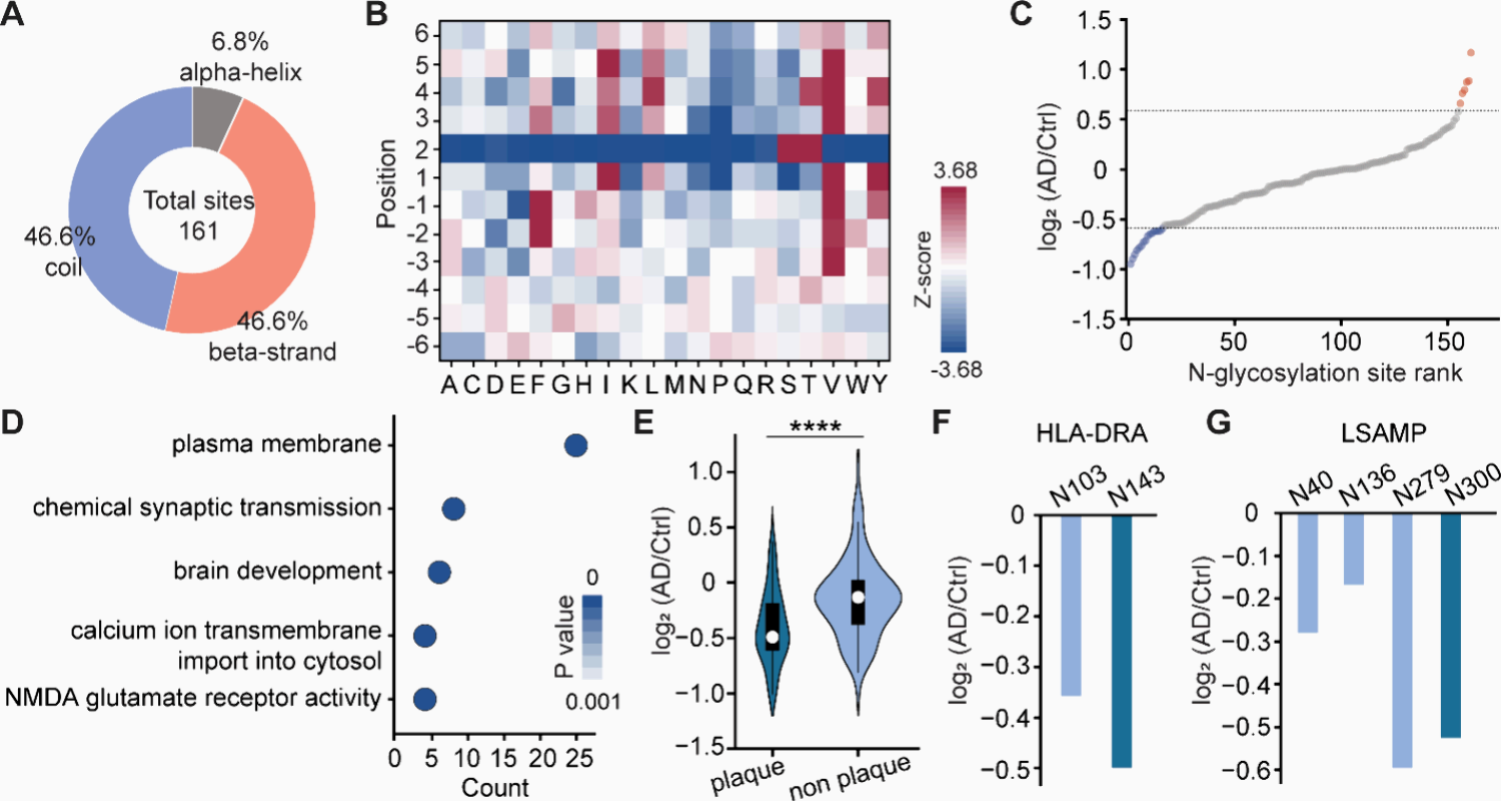
Site-specific analysis of N-glycosylation changes in APRs. (A) Comparison of the secondary structures of N-glycosylation sites located within APRs. (B) Occurrence of different amino acid residues next to N-glycosylation sites located within APRs, using all identified N-glycosylation sites as the background. The amino acids overrepresented in N-glycosylation sites located within APRs have positive Z-scores, while those reduced have negative ones. (C) Abundance changes of N-glycosylation sites located within APRs in AD. (D) Representative function annotations for glycoproteins with downregulated N-glycosylation sites. (E) Comparison of abundance changes of N-glycosylation sites located within APRs in plaque-associated glycoproteins to those within APRs but not plaque-associated glycoprotein. Interquartile ranges (IQRs) are shown as boxes, with the median as a white dot and the whiskers extending up to the most extreme points within 1.5-fold IQR. P values are assessed using the two-sided *t* test: * *P* < 0.05, ** *P* < 0.01, *** *P* < 0.001, **** *P* < 0.0001. (F) and (G) Site-specific analysis of HLA-DRA (F) and LSAMP (G). Dark blue indicates the N-glycosylation site located within APRs and prevents protein aggregation. Light blue indicates the N-glycosylation site located outside of APRs.

To further characterize these glycosylation sites, we examined the occurrence of different amino acid residues surrounding them, comparing their distribution to the background of all identified N-glycosylation sites (Figure 5B). It was found that hydrophobic amino acid residues such as phenylalanine (F), isoleucine (I), leucine (L), and valine (V) were overrepresented in the vicinity of N-glycosylation sites. Among the identified APR-associated glycosylation sites, only eight sites were upregulated, while 29 sites were downregulated, with the majority exhibiting no significant changes in AD (Figure 5C). Only a small subset of N-glycosylation sites within APRs are dysregulated in AD. GO analysis revealed that these downregulated N-glycosylation sites were primarily associated with biological processes including chemical synaptic transmission, brain development, calcium ion transmembrane import into the cytosol, and NMDA glutamate receptor activity, with most of these glycoproteins localized to the plasma membrane (Figure 5D). These findings suggest that the downregulation of N-glycosylation sites within APRs predominantly affects plasma-membrane proteins, thereby potentially disrupting chemical synaptic transmission in AD.

To investigate the role of N-glycosylation in protein aggregation, we extracted 45 N-glycosylation sites located within APRs of plaque-associated glycoproteins. When comparing the abundance changes of N-glycosylation sites within APRs in plaque-associated glycoproteins to those within APRs but not plaque-associated glycoproteins, we observed a significant decrease at the N-glycosylation level in plaque-associated glycoproteins (Figure 5E). This result indicates that certain proteins present or enriched in plaques may be driven by a reduction in N-glycosylation within APRs.^43^ For example, HLA has been reported to be associated with AD and enriched in plaques. In this study, we found that the N-glycosylation site at N143 of HLA-DRA, which is located within an APR, was significantly downregulated in AD, whereas the N-glycosylation site at N103 did not exhibit a marked decrease (Figure 5F). Additionally, LSAMP, a glycoprotein that mediates selective neuronal growth and axon targeting, exhibited altered glycosylation in AD. Among the four quantified N-glycosylation sites of LSAMP, the site at N300 within an APR was significantly downregulated in AD. The downregulation of N-glycosylation within APRs may contribute to protein aggregation and plaque formation in AD. Given the role of N-glycosylation in maintaining protein solubility and preventing aggregation, its dysregulation in AD may facilitate pathological protein deposition, ultimately exacerbating disease progression.

### Alterations in N-Glycosylation of Synaptic Proteins

Previous studies revealed dysregulation of synaptic proteins involved in calcium transport, neurotransmission, signaling, synaptic growth, and plasticity in AD.^44–46^ Given that many synaptic proteins are glycosylated. Using SynGO database,^47^ we quantified 942 N-glycosylation sites from 279 synaptic proteins (Figure 6A; Table S9). These glycoproteins are primarily involved in trans-synaptic signaling, regulation of synaptic assembly, synapse adhesion, and modulation of synaptic membrane potential (Figure 6B), highlighting the enrichment of synaptic membrane proteins in our data set. Most synaptic glycoproteins showed decreased abundance in AD brains (Figure 6C).

**Figure 6:**
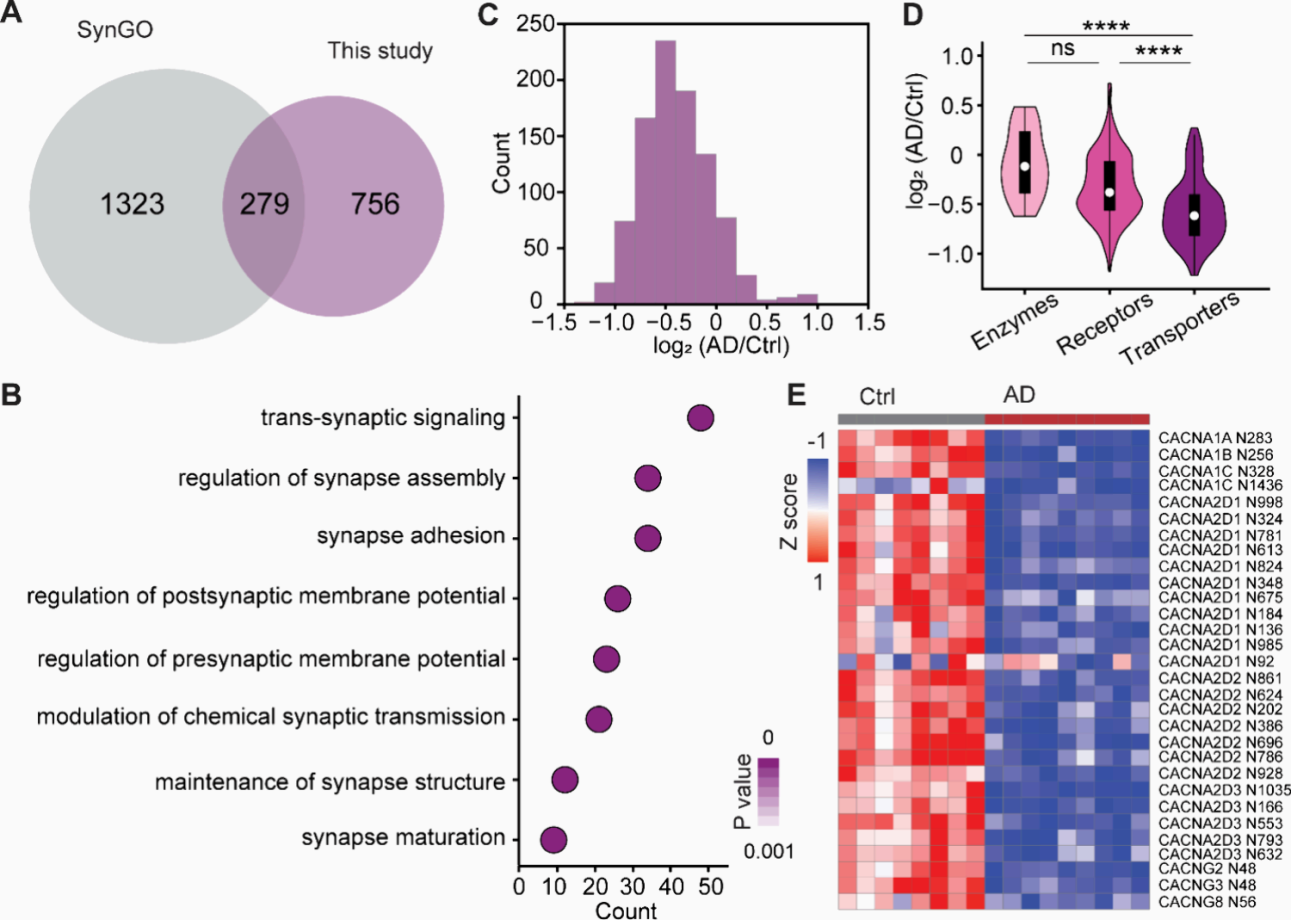
Site-specific analysis of synaptic glycoproteins. (A) Overlap of glycoproteins in this data set with the SynGO database. (B) SynGO biological process terms associated with synaptic glycoproteins. (C) Distribution of log_2_(AD/Ctrl) values for N-glycosylation sites on synaptic glycoproteins. (D) Comparison of N-glycosylation abundance changes among enzymes, receptors, and transporters. Interquartile ranges (IQRs) are shown as boxes, with the median as a white dot and the whiskers extending up to the most extreme points within 1.5-fold IQR. P values are assessed using the two-sided *t* test: * *P* < 0.05, ** *P* < 0.01, *** *P* < 0.001, **** *P* < 0.0001. (E) Heatmap showing alterations in N-glycosylation among Ca^2+^ ion channels.

Among protein classes, transporters exhibited the most significant decrease in N-glycosylation (Figure 6D). This finding suggests that transporters at the synapse are dramatically impaired in AD. To gain deeper insight into transporter-specific changes, we mapped N-glycosylation alterations in transporters at a site-specific level. Calcium ion plays a crucial role in neuronal function, and its dysregulation is a key feature of AD pathology.^48,49^ We identified 30 N-glycosylation sites from nine Ca^2+^ ion channels (Figure 6E), with an overall reduction in N-glycosylation compared to the controls. For example, CACNA2D1, a subunit of voltage-dependent calcium channels that regulate calcium current density, has 11 quantified N-glycosylation sites. Among them, N324, N613, N781, and N998 were significantly downregulated in AD. Given that N-glycosylation modulates the folding and activity of ion channels, the downregulation of N-glycosylation at these sites impairs the calcium ion concentration, ultimately affecting protein functions and cellular events required the calcium ion.

Besides the calcium ion channels, we observed an overall decrease in N-glycosylation of the GABA-gated chloride channels in AD (Figure S14). Notably, all three quantified N-glycosylation sites in GABRA3 were downregulated by more than 1.5-fold in AD. As GABA is the major inhibitory neurotransmitter in the brain, a reduction in N-glycosylation on the GABA-gated chloride channels could significantly disrupt Cl^–^ influx and neuronal depolarization.^50^ Furthermore, analysis of N-glycosylation changes in glutamate receptors revealed a widespread decrease in AD (Figure S15). Taken together, these findings provide a comprehensive map of N-glycosylation alterations in synaptic membrane proteins in AD, underscoring the potential impact of glycosylation dysregulation on synaptic function.

## Discussion

Protein N-glycosylation is known to modulate protein trafficking, receptor–ligand interactions, and ion channel activity,^8,51,52^ and aberrant glycosylation can significantly impact neuronal function. Our analysis revealed that glycoproteins involved in cholesterol efflux were upregulated in the AD brain tissues. Given the established link between lipid metabolism and AD pathology,^53,54^ altered glycosylation in cholesterol efflux may result in a disease-associated dysregulation of lipid homeostasis.

In this study, we comprehensively analyzed site-specific alterations of N-glycosylation in AD, revealing distinct patterns of glycosylation dysregulation across protein domains, aggregation-prone regions, and synaptic glycoproteins. Our findings provide critical insights into the molecular mechanisms of protein functions regulated by N-glycosylation in AD and highlight the potential roles of N-glycosylation in AD pathogenesis. The defined pathological features of AD are the accumulation of Aβ plaques and neurofibrillary tangles, which promote neurotoxicity and synaptic failure. We identified many N-glycosylation sites on plaque-associated proteins, some of which were significantly altered in AD.

Protein domains play critical roles in regulating the structures and interactions of glycoproteins, and N-glycosylation within specific domains may modulate protein interactions, trafficking, and stability. Our analysis revealed distinct regulations between N-glycosylation sites located within domains and those outside of any domain. Further analysis showed that many glycosylation sites located within functional domains were significantly altered in the AD brains, particularly in the ConA-like lectins/glucanases and Zn-dependent domains. Protein N-glycosylation has been implicated in regulating protein aggregation.^12,55^ We identified 161 N-glycosylation sites localized within aggregation-prone regions, which may interfere with protein–protein interactions and result in protein misfolding and aggregation. Notably, we found that several APR-associated N-glycosylation sites were significantly downregulated in AD, particularly on plaque-associated glycoproteins such as HLA-DRA and LSAMP. Given that glycosylation plays a key role in modulating protein solubility and aggregation,^12,55^ our findings suggest that the loss of N-glycosylation may contribute to the deposition of plaque-associated proteins.

Synaptic dysfunction is an early and critical event in AD progression,^31,33,34,36,44–46^ yet the molecular mechanisms contributing to synapse loss remain poorly understood. Our study revealed that N-glycosylation of synaptic glycoproteins is significantly dysregulated in AD, particularly among proteins involved in chemical synaptic transmission, trans-synaptic signaling, and synaptic adhesion. The majority of these synaptic glycoproteins exhibited reduced N-glycosylation levels, suggesting a potential impairment in their stability and function. In particular, transporters at the synaptic membrane showed the most pronounced decrease in N-glycosylation. Calcium ion channels, including CACNA2D1, exhibited significant reductions in site-specific N-glycosylation. Given that calcium ions are crucial for neuronal signaling,^48,49^ altered N-glycosylation in these channels may impact calcium homeostasis and disrupt synaptic signaling. Similarly, we observed the downregulation of N-glycosylation on GABA-gated chloride channels, such as GABRA3, which may disrupt inhibitory neurotransmission and contribute to synaptic imbalance in AD. Moreover, glutamate receptors exhibited widespread reductions in N-glycosylation, further implicating glycosylation loss in synaptic failure.

Collectively, in this work, we provide a comprehensive map of the alterations in N-glycosylation in AD brains, demonstrating that glycosylation changes are associated with protein aggregation, synaptic dysfunction, and impaired neurotransmission. These results not only offer insights into the roles of protein modifications in AD, but also highlight potential therapeutic targets for restoring synaptic function. Further investigation into the functional consequences of these N-glycosylation changes and their impacts on synaptic signaling will advance our understanding of AD pathophysiology and help identify novel strategies for disease intervention.

## Supplementary Material





















## Data Availability

The raw files have been deposited to the ProteomeXchange Consortium via the PRIDE partner repository with the data set identifier (Username: reviewer_pxd062640@ebi.ac.uk; Password: 7oGzRj79nkHS). The data that support the findings of this study are available in the Supporting Information of this article.

## Notes

Detailed protocols for tissue processing, glycopeptide enrichment using DBA, TMT labeling, LC-MS/MS acquisition, and bioinformatics analysis; supplementary figures showing numbers of N-glycoproteins, N-glycoproteome characterization, abundance changes of example glycoproteins, gene ontology analysis, and more other analysis results (PDF)
Table S1 - Human brain tissue sample information (XLSX)
Table S2 - Identification of glycoproteins in human brain tissues (XLSX)
Table S3 - Identified N-glycosylation sites from AD and control samples (XLSX)
Table S4 - Quantified glycoproteins in AD and control samples (XLSX)
Table S5 - Quantified glycopeptides from AD and control samples (XLSX)
Table S6 - NFT- or plaque-associated glycoproteins (XLSX)
Table S7 - Quantified N-glycosylation sites in human brains (XLSX)
Table S8 - Identified N-glycosylation sites located within APRs (XLSX)
Table S9 - Identified N-glycosylation sites from synaptic glycoproteins (XLSX)

## ¶

Incyte Corporation, Wilmington, Delaware 19803, United States

## ■

The Scripps Research Institute, La Jolla, California 92037, United States.
