# Supplementary material for: Comprehensive and Site-Specific Characterization of Protein N‑Glycosylation in AD Samples Reveals Its Potential Roles in Protein Aggregation and Synaptic Dysfunction
Source: Anal Chem. 2025 Oct 3;97(40):21873–82. doi: 10.1021/acs.analchem.5c02455 (PMC12529471; doi:10.1021/acs.analchem.5c02455)
Supplement: Supplementary file 1 [file ac5c02455_si_001.pdf]

Supporting Information

Comprehensive and site-specific characterization of protein N-glycosylation in AD samples reveals its potential roles in protein aggregation and synaptic dysfunction

Xing Xu[1#], Haiyan Tan[2#], Kejun Yin[1,6], Senhan Xu[1,7], Zeyu Wang[1], Geidy E. Serrano[3], Thomas G Beach[3], Xusheng Wang[4], Junmin Peng[5]*, Ronghu Wu[1]*

1 School of Chemistry and Biochemistry and the Petit Institute for Bioengineering and Bioscience, Georgia Institute of Technology, Atlanta, GA 30332, USA

2 Center for Proteomics and Metabolomics, St. Jude Children's Research Hospital, Memphis, TN 38105, USA

3 Banner Sun Health Research Institute, Sun City, AZ 85351, USA

4 Department of Neurology, University of Tennessee Health Science Center, Memphis, TN 38163, USA

5 Department of Structural Biology and Department of Developmental Neurobiology, St. Jude Children's Research Hospital, Memphis, TN 38105, USA

6 Present address: Incyte Corporation, Wilmington, DE 19803, USA

7 Present address: The Scripps Research Institute, La Jolla, CA 92037, USA

[#] Contributed equally

* Corresponding Authors: Junmin.peng@stjude.org (J.P.); ronghu.wu@chemistry.gatech.edu (R.W.)

Table of Contents

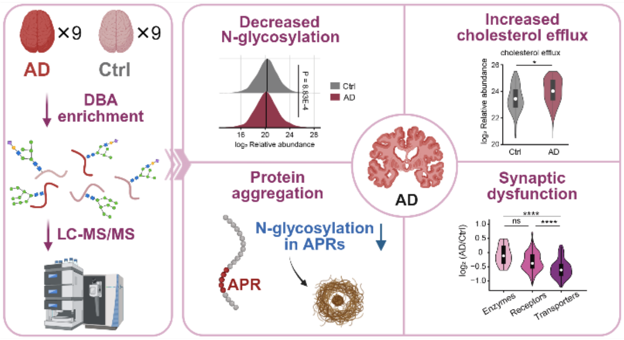

Experimental Section

Brain Sample Preparation and Protein Extraction

The samples were weighed and homogenized in the lysis buffer (50 mM HEPES, pH 8.5, 8.0 M urea, and 0.5% sodium deoxycholate, 100 µl buffer per 10 mg tissue) followed by protein concentration measurement using the BCA assay (Thermo Fisher) and then confirmed by the SDS gels stained with Coomassie. Quantified protein samples (~2 mg in the lysis buffer with 8 M urea) were purified by methanol/chloroform protein precipitation and resuspended in 400 µL of lysis buffer. The protein samples were further proteolyzed with Lys-C (Wako, 1:100 w/w) at 21 °C for 2 h, diluted by 4-fold to reduce urea to 2 M and continuously digested by trypsin (Promega, 1:50 w/w) at 21 °C overnight. The insoluble debris was kept in the lysates for the recovery of insoluble proteins. The digestion was terminated by the addition of 1% trifluoroacetic acid. After centrifugation, the supernatant was desalted using the Sep-Pak C18 cartridge (Waters) and then dried in Speedvac.

High-pH HPLC Fractionation and LC-MS/MS Analysis

The mixed TMT labeled sample was fractionated into 25 fractions using high-pH HPLC with a 40-min gradient (5-55% ACN) in 10 mM ammonium acetate (pH = 10). Each fraction was purified using the stage-tip method and dried again. Then, the sample was dissolved in 6 µL solvent with 5% ACN and 4% FA, and 4 µL of the solution were loaded by a Dionex WPS-3000TPLRS autosampler (UltiMate 3000 thermostatted Rapid Separation Pulled Loop Wellplate Sampler) onto a microcapillary column packed with C18 beads (Magic C18AQ, 1.9 µm, 200 Å, 75 µm x 16 cm,

Michrom Bioresources). Peptides were separated using a nanoflow reversed phase HPLC (Ultimate 3000 RSLCnano, Dionex). The buffer A was constituted with 0.125% formic acid and 2.5% ACN in water, and the buffer B contained 0.125% formic acid and 2.5% water in ACN. The microcapillary column was directly coupled for MS analysis using a Nanospray Flex ion source. The peptides were analyzed using an Orbitrap Exploris 480 mass spectrometer (Thermo). The samples were separated using a 95-min gradient of 5-30% buffer B at 0.3 μL/min. The full MS spectra were recorded using the following parameters: MS scan range: 350-1600 m/z; resolution: 120,000; Maximum injection time: Auto; AGC target: Standard. The precursor ions were selected for fragmentation by data-dependent acquisition (DDA). The most abundant precursor ions in each full MS scan were selected for fragmentation for 2 s at maximum. The following parameters were used for tandem MS scanning: Isolation width: 0.7 m/z; Isolation specificity: 90%; Resolution: 45,000; Normalized collision energy: 38%; AGC target: Standard; Max injection time: 100 ms. The selected ions were excluded for 45 s.

Protein and Glycopeptide Identification and Quantification

The raw file was converted to the mzXML format and searched using Comet [1] in the JUMPptm pipeline [2] against the human UniProt database (168,305 protein entries; downloaded April 2020) concatenated with a decoy database generated by reversing protein sequences to estimate false discovery rates. The following parameters were used for the search: 25 ppm precursor mass tolerance; 0.02 Da fragment ion tolerance; fully digested with trypsin; up to two missed cleavages; fixed modification: carbamidomethylation of cysteine (+57.0214), TMT labeling of the lysine residue (+304.2071) and the peptide N-terminus (+304.2071); variable modifications: oxidation of methionine (+15.9949), ^{18}O tag of asparagine (+2.9883, *N*-glycosylation site). The parameters

for whole proteome (WP) searching were the same except no ^{18}O tag of asparagine (+2.9883). The putative PSMs were further filtered by mass accuracy (±10 ppm) and then grouped by precursor ion charge state and filtered by matching scores to reduce the false discovery rates (FDR) to <1% for glycopeptides in glycosylation analysis or <1% FDR for proteins in whole proteome analysis. Protein and glycopeptide quantifications were performed based on the TMT reporter ions from MS2 using our previously optimized method.[3]

N-Glycosylation Site Localization by ModScore

The confidence of glycosylation site localization was estimated using ModScore.[4] All sites with ModScore > 13 (*P* value < 0.05) were considered as confidently localized. For site-specific analysis, all glycopeptides were further checked and only the sites with the consensus motif, i.e., NXS/T/C (X refers to any amino acid except proline), were kept. For site-specific quantification, only singly glycosylated peptides with well-localized sites were analyzed.

Bioinformatics Analysis

All analyses were performed using Excel, R and OriginLab 2022 unless mentioned otherwise. Differential analysis was performed using limma package in R.[5] Gene ontology (GO)-based clustering was performed using the Database for Annotation, Visualization and Integrated Discovery (DAVID, https://david.ncifcrf.gov/tools.jsp).[6-7] The potential pathogenicity of N-glycosylation sites was analyzed by AlphaMissense.[8] Domain analysis was performed by SUPERFAMILY 2.0 (https://supfam.org/).[9] The residue solvent accessibility and structure were predicted using NetsurfP (version 3.0).[10] Synaptic glycoproteins are extracted from SynGO

database (https://www.syngoportal.org/).[11] Heatmaps were performed using MORPHEUS (https://software.broadinstitute.org/morpheus/). The solubility data for NCAM2 glycoproteoforms was obtained from DQGlyco.[12]

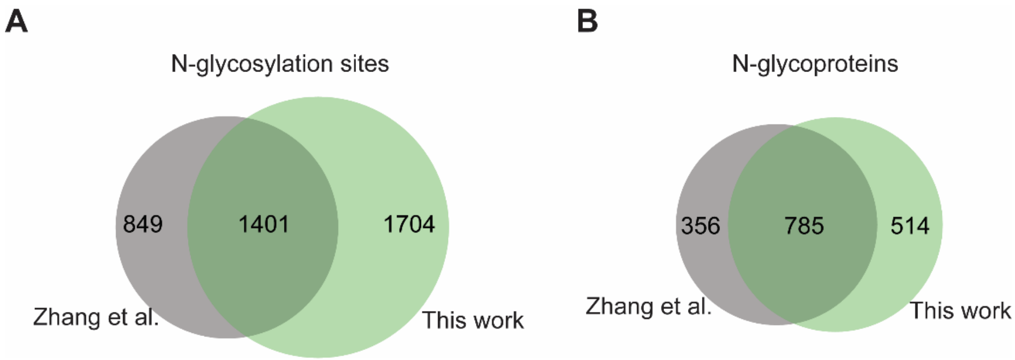

Figure S1. Overlap of N-glycosylation sites (A) and glycoproteins (B) identified in this study and those reported by Zhang *et al*.

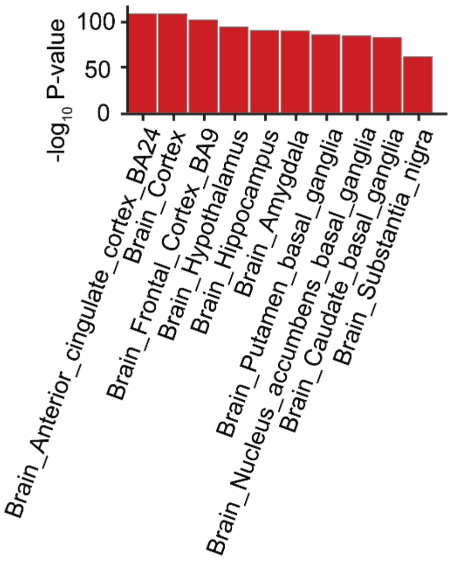

Figure S2. Tissue expression of all identified glycoproteins based on FUMA GTEx V8 analysis.

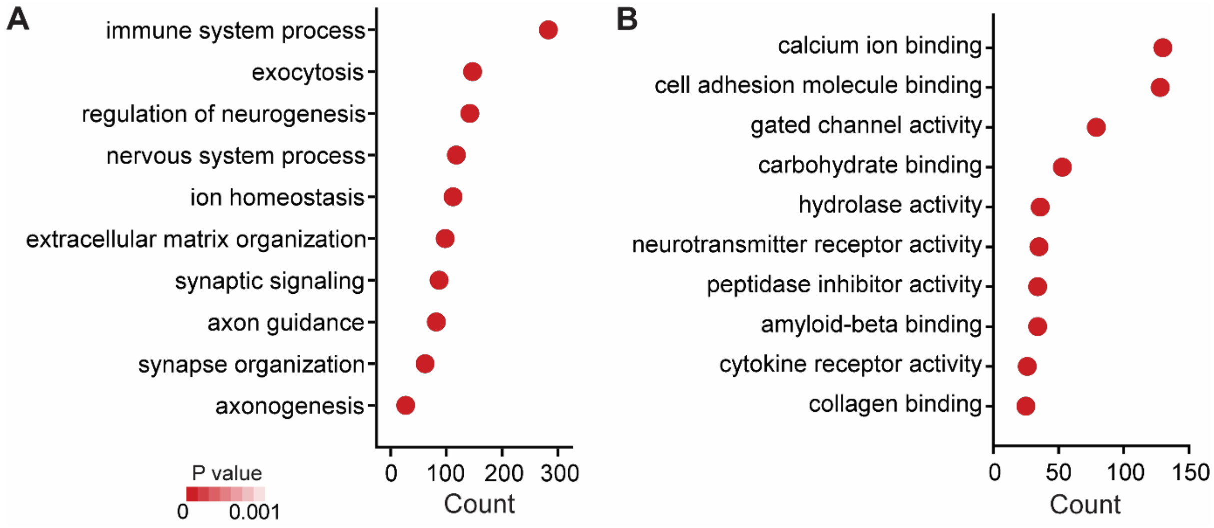

Figure S3. Gene ontology clustering of identified glycoproteins based on biological processes (A) and molecular functions (B).

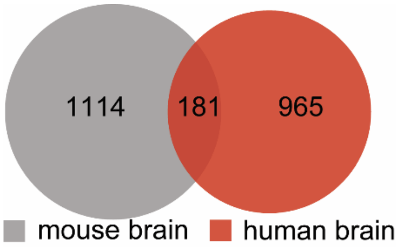

Figure S4. Comparison of the N-glycoproteome between the mouse and human brain tissues.

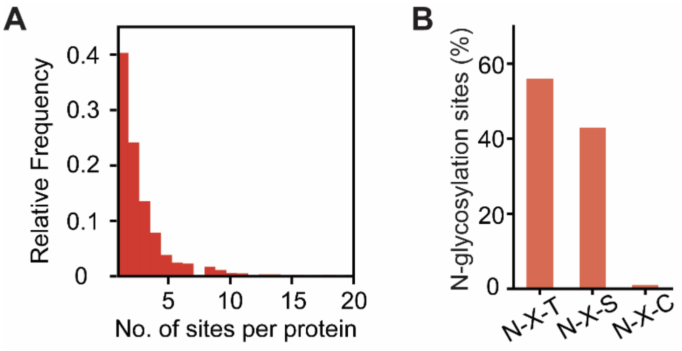

Figure S5. Distribution of the number of N-glycosylation sites per protein (A). Distribution of identified N-glycosylation sites based on the consensus sequons (B).

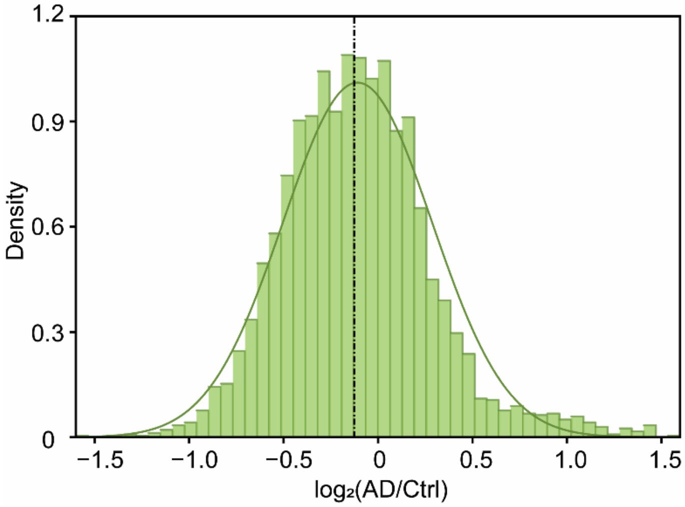

Figure S6. Density distribution of log2 fold changes of glycopeptides in AD brains.

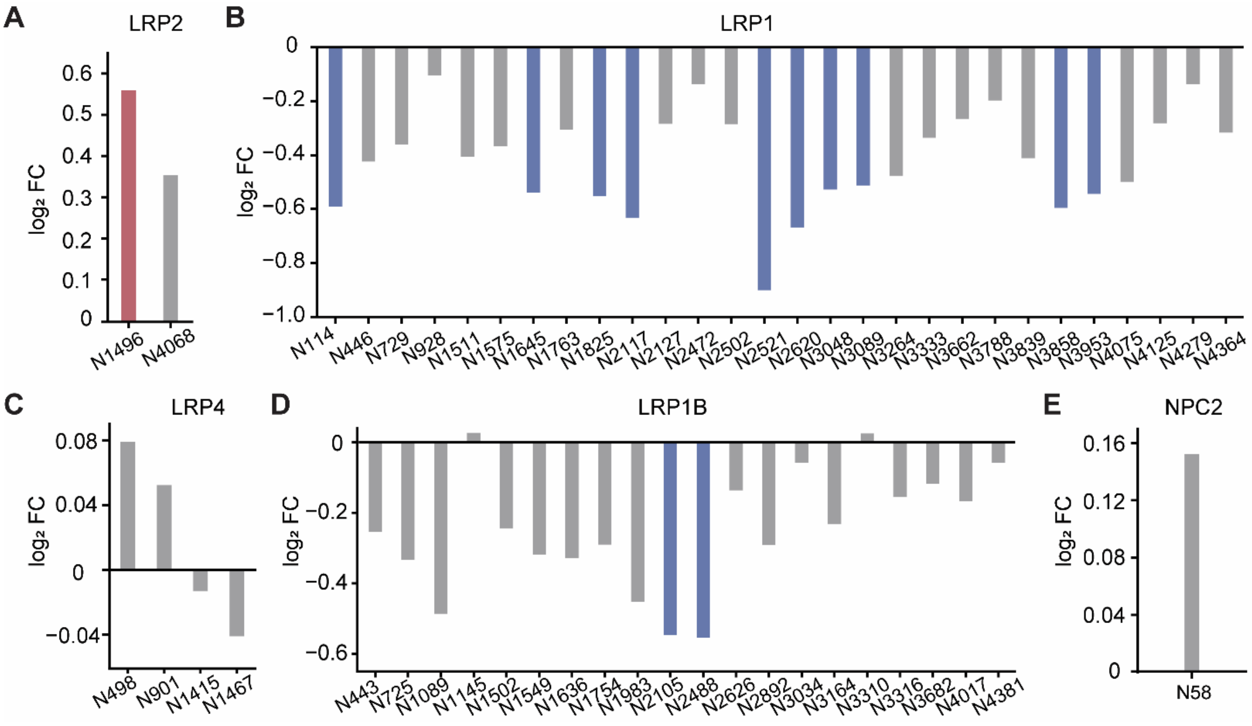

Figure S7. Abundance changes of glycosylation sites of cholesterol regulators. (A) LRP2. (B) LRP1. (C) LRP4. (D) LRP1B. (E) NPC2.

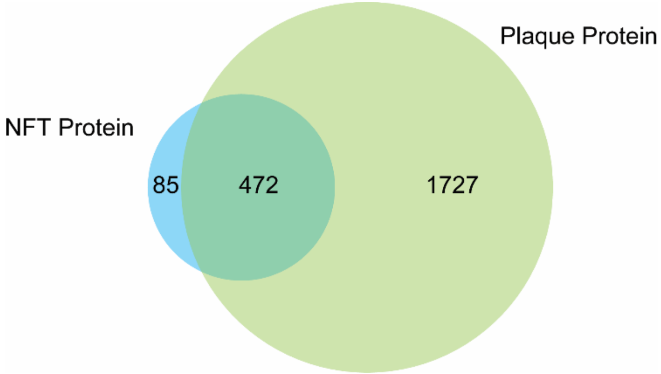

Figure S8. Overlap of proteins present or enriched in neuropathological lesions in AD.

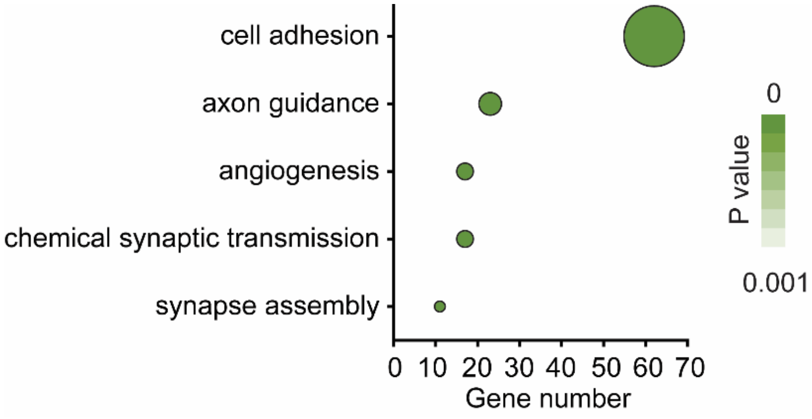

Figure S9. Gene ontology analysis of all plaque-associated glycoproteins based on biological process.

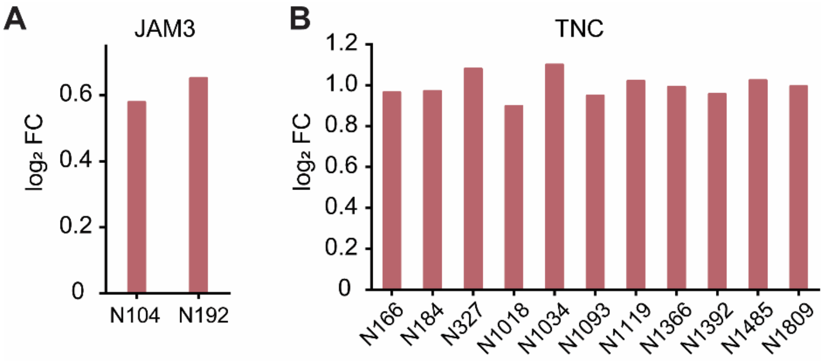

Figure S10. All glycosylation sites that were upregulated in JAM3 (A) and TNC (B).

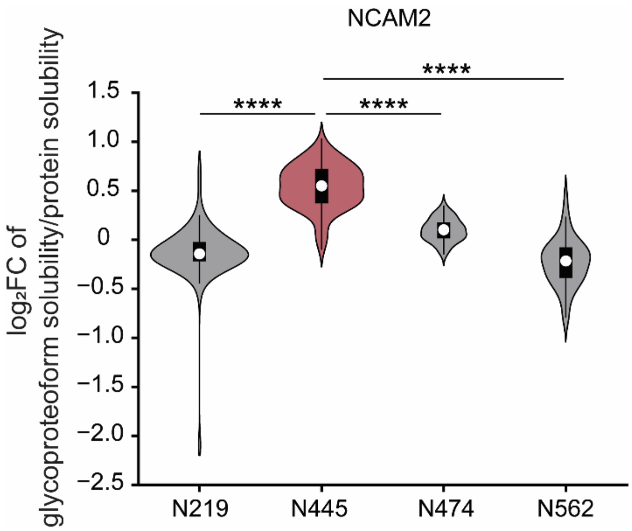

Figure S11. Comparison of NCAM2 glycoproteoform solubility at different N-glycosylation sites. Interquartile ranges (IQRs) are shown as boxes, with the median as a white dot and the whiskers extending up to the most extreme points within 1.5-fold IQR. P values are assessed using the two-sided t test: * P < 0.05, ** P < 0.01, *** P < 0.001, **** P <0.0001.

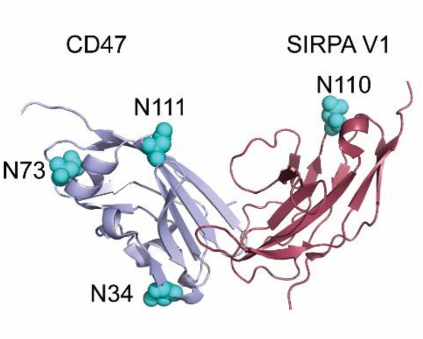

Figure S12. Structure of CD47-SIRPA v1 (PDB: 4CMM). Glycosylated asparagine residues are highlighted as cyan spheres.

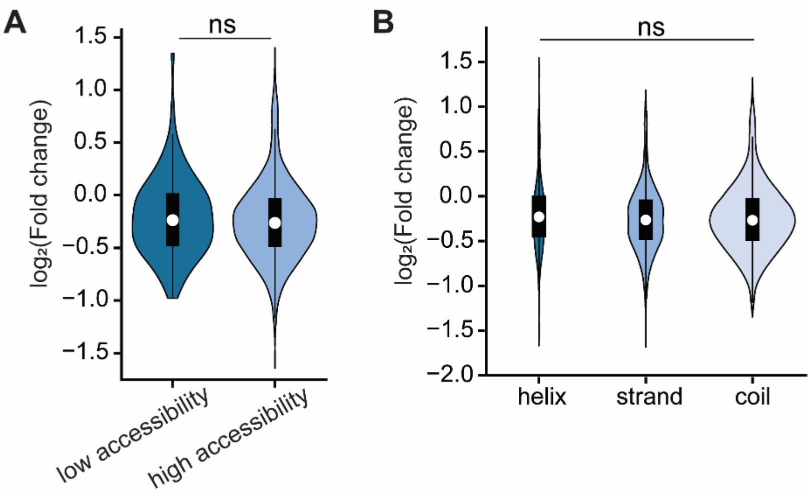

Figure S13. Comparison of abundance changes of N-glycosylation sites with low or high solvent accessibility (A) and different types of secondary protein structures (B). Interquartile ranges (IQRs) are shown as boxes, with the median as a white dot and the whiskers extending up to the most extreme points within 1.5-fold IQR. P values are assessed using the two-sided t test.

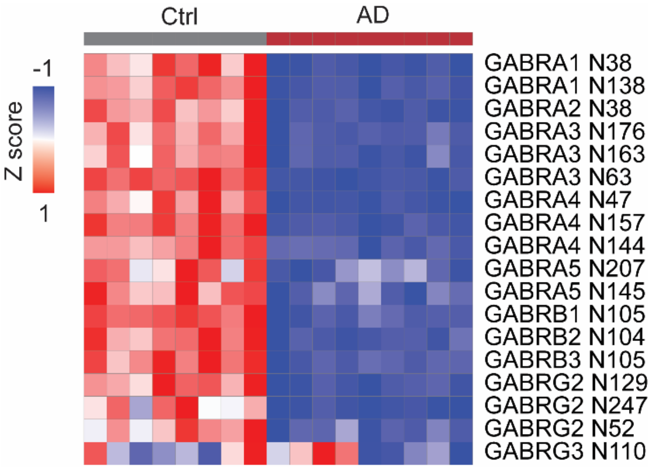

Figure S14. Heatmap of N-glycosylation changes in GABA-gated chloride channels between AD and control samples.

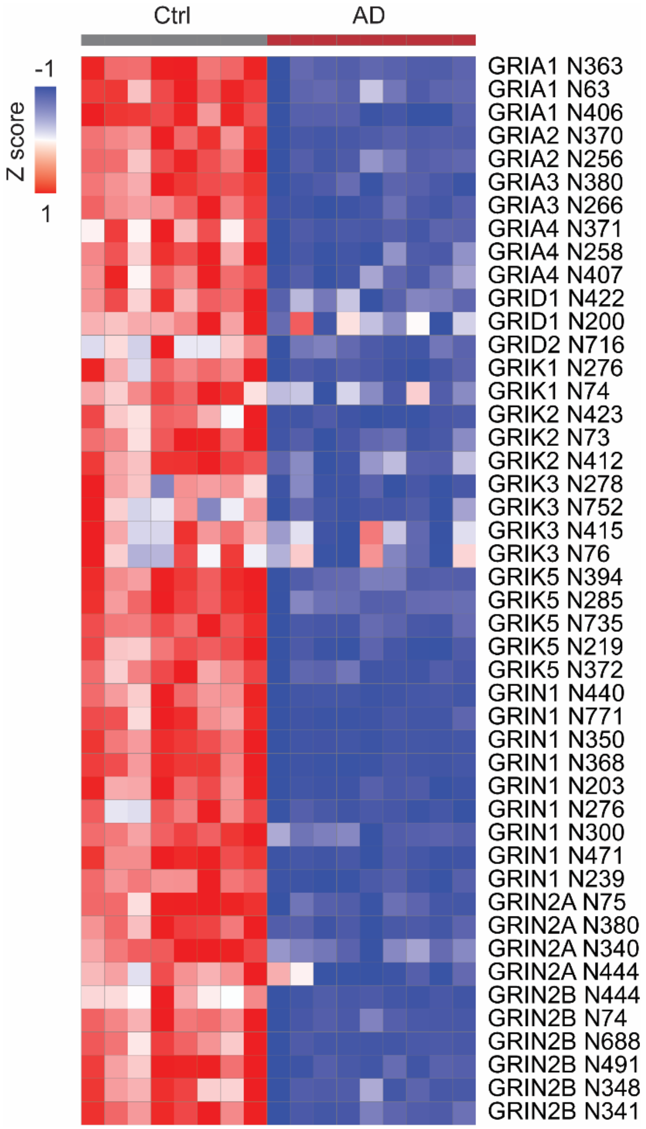

Figure S15. Heatmap of N-glycosylation changes in glutamate receptors.

References

(1) Eng, J. K.; Jahan, T. A.; Hoopmann, M. R. Comet: An open-source MS/MS sequence database search tool. *Proteomics* **2013**, *13* (1), 22-24.

(2) Poudel, S.; Vanderwall, D.; Yuan, Z.-F.; Wu, Z.; Peng, J.; Li, Y. JUMPptm: Integrated software for sensitive identification of post-translational modifications and its application in Alzheimer's disease study. *Proteomics* **2023**, *23* (3-4), 2100369.

(3) Niu, M.; Cho, J.-H.; Kodali, K.; Pagala, V.; High, A. A.; Wang, H.; Wu, Z.; Li, Y.; Bi, W.; Zhang, H.; et al. Extensive peptide fractionation and y1 ion-based interference detection method for enabling accurate quantification by isobaric labeling and mass spectrometry. *Anal. Chem.* **2017**, *89* (5), 2956-2963.

(4) Beausoleil, S. A.; Villén, J.; Gerber, S. A.; Rush, J.; Gygi, S. P. A probability-based approach for high-throughput protein phosphorylation analysis and site localization. *Nat. Biotechnol.* **2006**, *24* (10), 1285-1292.

(5) Ritchie, M. E.; Phipson, B.; Wu, D.; Hu, Y.; Law, C. W.; Shi, W.; Smyth, G. K. limma powers differential expression analyses for RNA-sequencing and microarray studies. *Nucleic Acids Res.* **2015**, *43* (7), e47-e47.

(6) Sherman, B. T.; Hao, M.; Qiu, J.; Jiao, X.; Baseler, M. W.; Lane, H. C.; Imamichi, T.; Chang, W. DAVID: a web server for functional enrichment analysis and functional annotation of gene lists (2021 update). *Nucleic Acids Res.* **2022**, *50* (W1), W216-W221.

(7) Huang, D. W.; Sherman, B. T.; Lempicki, R. A. Systematic and integrative analysis of large gene lists using DAVID bioinformatics resources. *Nat. Protoc.* **2009**, *4* (1), 44-57.

(8) Cheng, J.; Novati, G.; Pan, J.; Bycroft, C.; Žemgulytė, A.; Applebaum, T.; Pritzel, A.; Wong, L. H.; Zielinski, M.; Sargeant, T.; et al. Accurate proteome-wide missense variant effect prediction with AlphaMissense. *Science* **2023**, *381* (6664), eadg7492.

(9) Pandurangan, A. P.; Stahlhacke, J.; Oates, M. E.; Smithers, B.; Gough, J. The SUPERFAMILY 2.0 database: a significant proteome update and a new webserver. *Nucleic Acids Res.* **2019**, *47* (D1), D490-D494.

(10) Høie, M. H.; Kiehl, E. N.; Petersen, B.; Nielsen, M.; Winther, O.; Nielsen, H.; Hallgren, J.; Marcatili, P. NetSurfP-3.0: accurate and fast prediction of protein structural features by protein language models and deep learning. *Nucleic Acids Res.* **2022**, *50* (W1), W510-W515.

(11) Koopmans, F.; van Nierop, P.; Andres-Alonso, M.; Byrnes, A.; Cijsouw, T.; Coba, M. P.; Cornelisse, L. N.; Farrell, R. J.; Goldschmidt, H. L.; Howrigan, D. P.; et al. SynGO: An evidence-based, expert-curated knowledge base for the synapse. *Neuron* **2019**, *103* (2), 217-234.

(12) Potel, C. M.; Burtscher, M. L.; Garrido-Rodriguez, M.; Brauer-Nikonow, A.; Becher, I.; Le Sueur, C.; Typas, A.; Zimmermann, M.; Savitski, M. M. Uncovering protein glycosylation dynamics and heterogeneity using deep quantitative glycoprofiling (DQGlyco). *Nat. Struct. Mol. Biol.* **2025**, 1-16.
